# A Lateral Flow Assay for the Detection of *Leptospira lipL32* Gene Using CRISPR Technology

**DOI:** 10.3390/s23146544

**Published:** 2023-07-20

**Authors:** Satheesh Natarajan, Jayaraj Joseph, Balamurugan Vinayagamurthy, Pedro Estrela

**Affiliations:** 1 Healthcare Technology Innovation Centre, Indian Institute of Technology Madras, Chennai 600113, India; 2 Department of Electrical Engineering, Indian Institute of Technology Madras, Chennai 600036, India; jayaraj@ee.iitm.ac.in; 3 Indian Council of Agricultural Research-National Institute of Veterinary Epidemiology and Disease Informatics (ICAR-NIVEDI), Bangalore 560064, India; b.vinayagamurthy@icar.gov.in; 4 Department of Electronic and Electrical Engineering, University of Bath, Bath BA2 7AY, UK; p.estrela@bath.ac.uk; 5 Centre for Bioengineering & Biomedical Technologies, University of Bath, Bath BA2 7AY, UK

**Keywords:** *Leptospira*, CRISPR, dFnCas9, lateral flow device

## Abstract

The clinical manifestation of leptospirosis is often misdiagnosed as other febrile illnesses such as dengue. Therefore, there is an urgent need for a precise diagnostic tool at the field level to detect the pathogenic *Leptospira lipL32* gene at the molecular level for prompt therapeutic decisions. Quantitative polymerase chain reaction (qPCR) is widely used as the primary diagnostic tool, but its applicability is limited by high equipment cost and the lack of availability in every hospital, especially in rural areas where leptospirosis mainly occurs. Here, we report the development of a CRISPR dFnCas9-based quantitative lateral flow immunoassay to detect the *lipL32* gene. The developed assay showed superior performance regarding the lowest detectable limit of 1 fg/mL. The test is highly sensitive and selective, showing that leptospirosis diagnosis can be achieved with a low-cost lateral flow device.

## 1. Introduction

Early and accurate detection of pathogens is crucial for promptly administering therapeutic decisions. The most common zoonotic disease in humans is leptospirosis, caused by the pathogenic bacteria *Leptospira*, with a million cases reported yearly, which includes 60,000 deaths [1,2]. The clinical manifestations of human leptospirosis range from subclinical infection to severe fatal disease [2,3]. The symptoms are not disease-specific, so laboratory confirmation is required to diagnose leptospirosis [4]. Though the microscopic agglutination test (MAT) is the gold standard method, serological complications, time consumption, and live strain maintenance requirements have made this method less than ideal [5]. Due to their complicated isolation methodologies, slow growth, low recovery, and high contamination, the culture methods also make them unsuitable for widespread clinical diagnosis [6]. Immunofluorescence assays targeting *OmpL54* can also be used [7], but they provide many false-negative results [8].

Although molecular techniques such as real-time polymerase chain reaction (qPCR) are the most validated diagnostic methods for genes such as *lipL32* [9] and *lfb1* [10], they fail the recognition of the species from “intermediate” clade or pathogenic *Leptospira* spp. group II clade [11,12]. This issue has been suggested as a potential factor contributing to the under-reporting of *Leptospira* spp. in several geographic regions [11]. To overcome this problem, precise diagnostic tools are proposed and developed in this work to detect nucleic acids utilizing genome editing techniques such as CRISPR-Cas9 endonucleases [13]. Here, we applied and optimized inactive *Francisella novicida* Cas9 (dFnCas9)-based technology [14] to diagnose the pathogenic biomarker *lipL32* gene for *Leptospira* detection. These tools use the specific properties of the Cas enzymes, i.e., upon recognition of the foreign pathogenic DNA, the non-specific DNase/RNase activity of the Cas enzyme activity will be stimulated for its recognition of the foreign DNA. It is also a rapid, cost-effective method for nucleic acid detection in clinical samples with excellent sensitivity and specificity, making them an ideal tool for point-of-care (POC) diagnosis [15] and, in recent years, the method has been implemented with lateral flow assays [16,17,18].

The dFnCas9 has a very high intrinsic specificity to the target [14]; we speculated that the enzyme could also be used for detecting the *lipL32* gene on a paper strip with high accuracy. Herein, we demonstrate a dFnCas9-based quantitative lateral flow assay to detect the *lipL32* gene. Upon DNA loading, an automatic sandwiched assay was completed within 15 min, allowing for ultra-specific assay compared to other sensors enabling on-field analysis or bedside testing. The lateral flow paper strip was used on the quantitative strip reader iQuant developed at the Healthcare Technology Innovation Centre, Indian Institute of Technology, Madras, India [19,20,21]. The advantages of the reader and performance comparison with other systems can be found in the literature [19].

**Figure 1:**
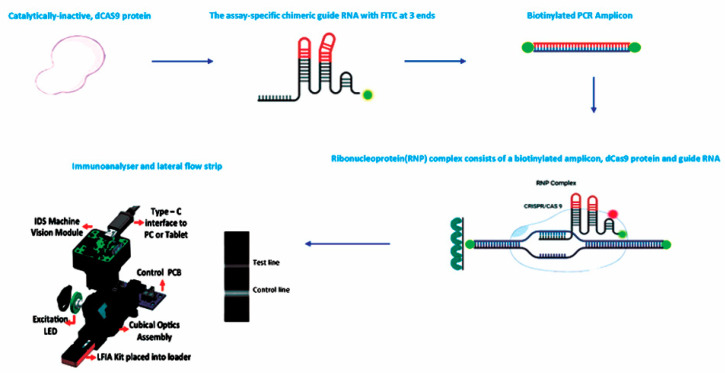
Experimental flow diagram starting from the dFnCas9 to the lateral flow assay.

We explored the dFnCas9 with PCR amplification and lateral flow assay for cost-effective *Leptospira* detection with high sensitivity and avoided the need for sophisticated instrumentation. To enable such diagnosis, we used our quantitative lateral flow assay strip to capture RNP-bound biotinylated substrate molecules on a test line coated with the streptavidin of the paper strip using FITC-labelled chimeric gRNA (Figure 1). To make the strip readout reproducible in point-of-care settings, we chemically modified the chimeric gRNA using synthetic backbone modifications (phosphorothioate) to increase the stability and robustness of the readout. In the conjugation pad, we conjugated the FITC antibodies with the Alexafluor-674 to make the kit quantitative. Finally, using an optimized PCR protocol followed by dFnCas9, we developed an assay to detect *Leptospira lipL32* gene sequences from DNA samples within an hour.

## 2. Materials and Methods

Nitrocellulose membranes (HiFlow135) were procured from Merck Millipore (Bedford, MA, USA). Sample pad (CF-4), conjugation pad (Standard-17), and absorbent pad (CF-6) were obtained from Cytiva, UK. The anti-FITC monoclonal antibodies, goat anti-chicken IgY antibody (AF 010), and normal chicken IgY control (AB-101-C) were bought from R&D Systems (Minneapolis, MN, USA). Biotin-BSA, Streptavidin, and Alexa Fluor 647 NHS ester were procured from Thermo Fisher (Waltham, MA, USA); Sephadex G20 column from GE Healthcare (Uppsala, Sweden). PBS (137 mm NaCl, 2.7 mm KCl, 10 mm Na_2_HPO_4_, 1.8 mm KH_2_PO_4_) and PB (75.4 mm Na_2_HPO_4_·7H_2_O, 24.6 mm NaH_2_PO_4_·H_2_O) buffers, NaOH, NaHCO_3_, NaN_3_, BSA, and Tween-20, were purchased from Sigma-Aldrich (St. Louis, MO, USA).

### 2.1. Study Design

The study intended to develop robust CRISPR diagnostics that can perform with high accuracy for the *lipL32* gene of the *Leptospira* at a significantly low cost and time. For the *lipL32* gene detection using *Leptospira* positive DNA extracted from a reference, *Leptospira interrogans* serovar Icterohaemorrhagiae was received from the National Institute of Veterinary Epidemiology and Diseases Informatics, Bangalore. 

### 2.2. dFnCas9 Protein

The plasmids containing dFnCas9 (catalytically inactive, dead) [14] were kindly gifted by Dr. Debojyoti Chakraborty, IGIB, New Delhi. The sequences specific to the protein were transformed and expressed in *Escherichia coli* Rosetta 2 (DE3) (Novagen). Rosetta 2 (DE3) cells were cultured in Luria Bertani (LB) broth medium (with 50 mg/mL kanamycin) at 37 °C and induced using 0.5 mM Isopropyl β-D-thiogalactopyranoside (IPTG) when OD_600_ reached 0.6. After overnight culturing, *E. coli* cells were harvested and resuspended in a lysis buffer (20 mM HEPES, 500 mM NaCl, 5% glycerol) supplemented with 1× PIC (Roche) containing 100 mg/mL lysozyme. After cell lysis by sonication, the lysate was put through Ni-NTA beads (Roche). The eluted protein was purified by Superdex 200 16/60 column (GE Healthcare) in a buffer solution of 20 mM HEPES pH 7.5, 150 mM KCl, 10% glycerol, and 1 mM DTT. The purified proteins were quantified by a BCA protein assay kit (Thermo Fisher Scientific, Waltham, MA, USA) and stored at −80 °C until further use.

### 2.3. LipL32 Gene Detection PCR

The *lipL32* gene regions were PCR amplified using 5′ biotin-labelled primers. The chimeric gRNA (crRNA: TracrRNA) was prepared by mixing the crRNA (*lipL32* gene) and the synthetic 3′-FITC-labelled TracrRNA in an equimolar ratio with the help of the annealing buffer (100 mM NaCl, 50 mM Tris-HCl pH 8, and 1 mM MgCl_2_), and heated at 95 °C for 2–5 min and then allowed to cool at room temperature for 15–20 min. The chimeric gRNA-dead FnCas9 RNP complex was prepared by mixing it equally (protein:sgRNA molar ratio, 1:1) in buffer (20 mM HEPES, 150 mM KCl, 1 mM DTT, 10% glycerol, 10 mM MgCl_2_) and incubating for another 10 min at room temperature. The target biotinylated amplicons were then incubated in the RNP complexes for 10 min at 37 °C. The detailed protocol for the CRISPR/CAS and the PCR for *lig A*, *lig B*, and *lipL41* are also given in the supplementary file.

### 2.4. Antibody Conjugation

The monoclonal mouse anti-FITC antibody and the anti-chicken IgY were conjugated with the fluorescent organic dye Alexa Fluor 647, which contains a succinimidyl ester to react with the primary amines present in the antibodies. Briefly, the detection antibody, both monoclonal mouse anti-FITC antibody and the anti-chicken IgY @ 1 mg/mL in 10 mM phosphate solution (pH 7.4), was mixed with the Alexa Fluor 647 (20 molar excess) for 1 h at RT. The conjugates were purified by Sephadex G20 gel chromatography column by centrifuging at 4000 rpm for 5 min and collecting the labelled antibody in 1× PBS buffer containing 1% BSA, 0.1% Tween 20. The conjugate was mixed with glycerol to the final concentration of 10 µg/mL and stored at 4 °C until further use.

### 2.5. Lateral Flow Immunoassay (LFIA)

The LFIA strip contains a sample pad (10 mm length), a polyester fiber conjugate pad (13 mm length), a nitrocellulose membrane (25 mm length), and an absorbent pad (15 mm length). The sample pad was pre-treated with sample pad pre-treatment buffer containing (1× PBS, 5% BSA, 0.1% Tween-20), followed by air drying at 1 h at RT. The conjugate pad, after pre-treatment, was dispensed with 0.3 ng/mL antibody-dye conjugate in 10 mm PBS buffer with 0.1% Tween, 0.1% BSA and subsequently dried for 1 h at 40 °C. The NC membrane was dispensed with 0.5 mg/mL of streptavidin, and 0.2 mg/mL anti-chicken IgY was dispensed over the analytical strips at a 0.6 µL/cm rate using the Claremont antibody dispenser with the chemryx syringe pump in the Test and Control lines at the rate of 1 µL/cm. Following dispensing, the analytical NC membrane was airdried at 37 °C for 12 h. Finally, all the pads were laminated with a partial overlapping of 2 mm and cut with a width of 3.1 mm with the Werfen guillotine cutter. The assembled LFIA strips were stored at 4 °C until further use.

This experiment was designed to investigate the CRISPR-based dFnCas9 detection of the *Leptospira lipL32* gene using the lateral flow immunoassay strip, which involved dispensing capture biomolecules onto the test lines of the NC membrane, the addition of Alexa-labelled biomolecules on to the conjugate pads, cartridge assembly, and running of samples containing with the adequate sample biomolecules. The typical test procedure involved the addition of 90 µL of the appropriate lateral flow chase buffer to the sample pad of the LFIA cartridge, which was subsequently inserted into the iQuant analyzer [21]. The process was monitored for about 15 min. The LED-based instrument scanned the signal generated in the Nitro Cellulose membrane as a two-dimensional pixel map for the quantification of the Alexa Fluor 647 labelled component. The pixel map was processed by the NI LabView software [20]. All the quantitative data were assessed by GraphPad Prism 6.0 (GraphPad Software, La Jolla, CA, USA). The intensity data were used for the calculation of the pixel volume of the test, V_T_, and control, V_C_, lines [20]. The mean volume ratio, V_R_, defined as the ratio V_T_/V_C_, was also calculated when required. The immunoassays were performed in triplicate for each of the samples.

### 2.6. Leptospira Standards and Samples

The calibration curves were constructed for the range of 0–10 pg/mL concentration using *Leptospira* standards prepared in 1× PBS, 1% BSA, and 0.1% Tween-20. After the analysis, the mean volume ratio, V_r_, was plotted vs. the *lipL32* gene standard concentration to construct the calibration curves. The standard deviation was utilized to calculate the coefficient of variation (CoV) according to CoV = SD/mean × 100%. The calibration curve data were analyzed by linear regression, and the limits of detection (LOD) and quantitation (LOQ) were determined based on the residual standard deviation and slope of the calibration curves obtained. Standard samples with the same concentrations were also prepared in the buffer. These diluted samples were then analyzed for LFIA.

## 3. Results and Discussion

### 3.1. LFIA and Fluorescence Assay Validation with lipL32 Gene Leptospira Genomic DNA

Genomic DNA from the reference strain *Leptospira* interrogans serovar Icterohaemorrahae was diluted for the PCR with the designated primer employed for the *lipL32* gene (Table 1).

**Figure 2:**
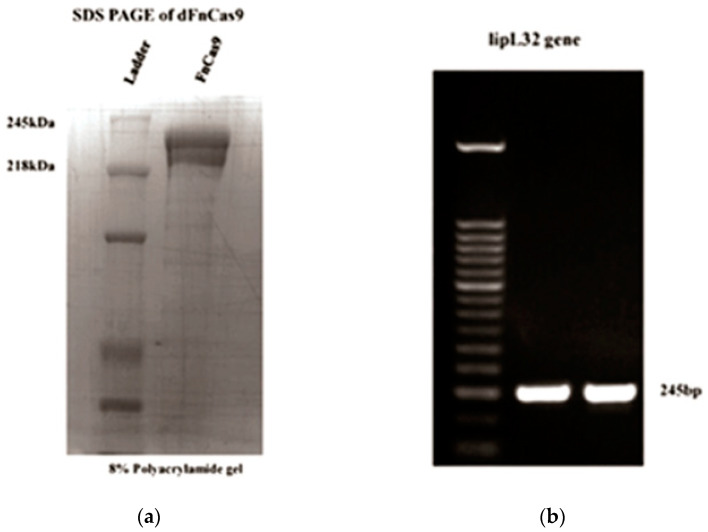
(**a**) SDS PAGE of purified en31dFnCas9; (**b**) PCR results for the *Leptospira lipL32* gene.

**Figure 3:**
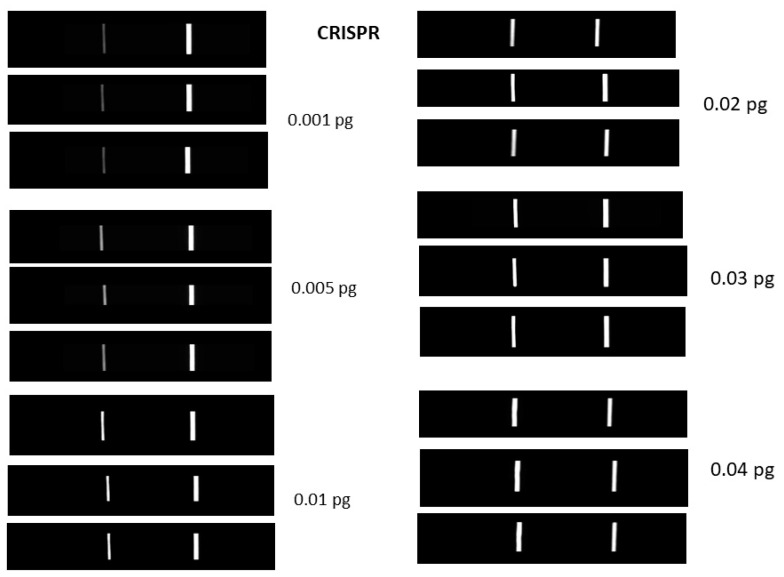
Pictures of the lateral flow devices for target DNA in the concentrations 0.001, 0.005, 0.01, 0.02, 0.03, and 0.04 pg/mL. Three independent devices are shown for each concentration.

Figure 2 shows the purified protein dFnCas9 on the SDS page and the gel electrophoresis pictures of the *lipL32* gene. This amplicon was then used to establish the limit of detection (LOD) of the LFIAs. The fluorescence signal above the background was observed for each of the samples, indicating that the dFnCas9-based assay limit of detection (LOD) was similar to that of the RT-PCR. We further quantified the *lipL32* gene amplicons and performed a copy number LOD. Fluorescence above the background was observed with 0.001 pg/mL copies of target DNA (Figure 3). Under the 35-cycle PCR amplification parameters, this represents the copies less than 5 are the starting material. LFIA was optimized with the soak DNA and irrelevant sgRNA. Under these conditions, test lines were observable at a sensitivity that was below that of RT-PCR or Cas9 fluorescence. These results showed that the LFIA with Cas9 fluorescence can detect the nucleic acid from the *Leptospira lipL32* gene.

### 3.2. Optimization

**Figure 4:**
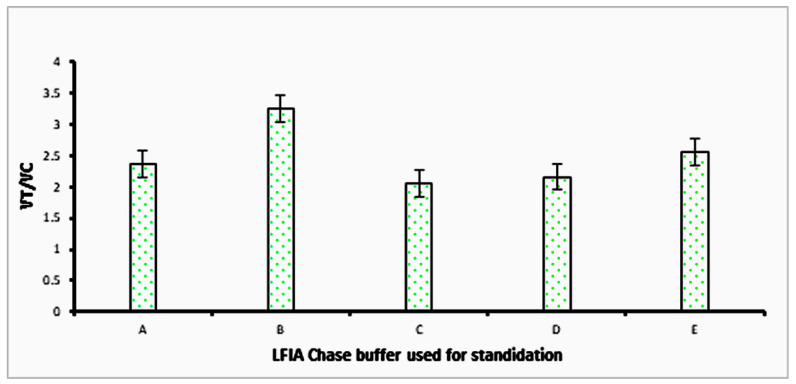
Performance of different mixtures of chase buffer. A: PBS + 0.75% BSA + 0.75% Tween 20 + 1% PVP40 + 1mm EDTA; B: PBS + 0.5% BSA + 0.5% Tween 20 + 2% PVP40 + 5mm EDTA; C: PBS + 1.25% BSA + 1% Tween 20 + 1.5% PVP40 + 3mm EDTA; D: PBS + 1.25% BSA + 1.25% Tween 20 + 1.5% PVP40 + 2mm EDTA; E: PBS + 1.75% BSA + 1.5% Tween 20 + 2.5% PVP40 + 2mm EDTA.

**Figure 5:**
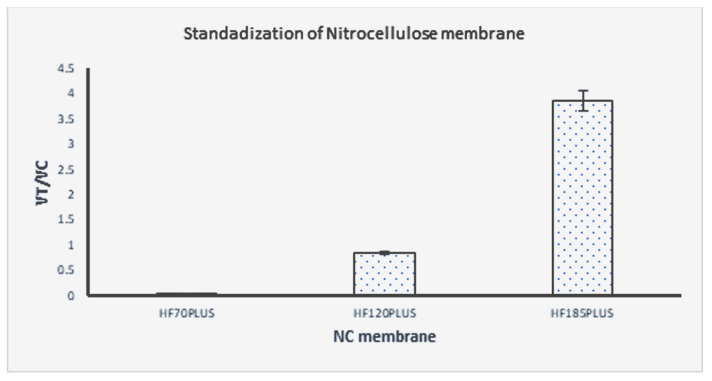
Effect of the different NC membranes on the signal: HF70 Plus, HF120 Plus, and HF180 Plus.

In the present work, several parameters impacting the performance of the *Leptospira* test strip (including the concentration of lateral flow assay chase buffer and NC membrane) were evaluated to obtain the maximal sensing efficacy. Details of the assay optimization are presented in Figure 4 and Figure 5. The highest peak signal of V_T_/V_C_ indicates the best conditions. The chase buffer with PBS + 0.5% BSA + 0.5% Tween 20 + 2% PVP-40 + 5 mm EDTA yielded the best results, as seen in Figure 4, and hence was used in the subsequent experiments. Different NC membranes were tested using this buffer with NC HF180 giving the best results (Figure 5). This membrane was then used for all subsequent experiments.

### 3.3. Analytical Performance

**Figure 6:**
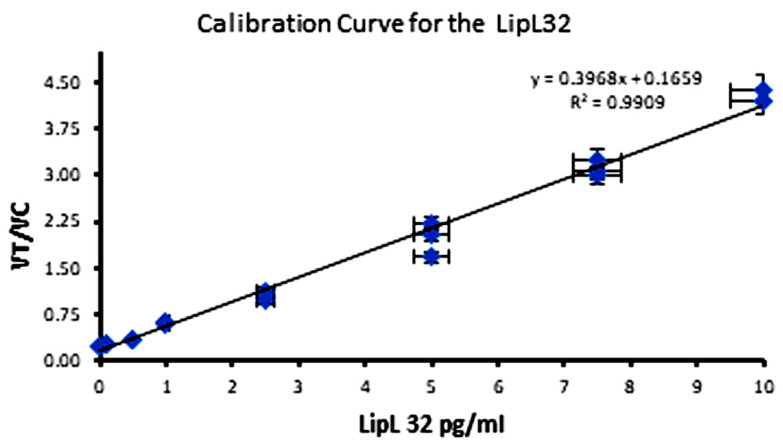
Calibration curve of the dFnCas9 LFIA in the concentration range 0–10 pg/mL. Linear fit shows an R^2^ value of 0.9909.

The performance of the CRIPSR-based dFnCas9 lateral flow assay for detecting the *lipL32* gene was evaluated by the quantitative immunoanalyzer iQuant developed by our institute (HTIC, IIT Madras). As seen in Figure 6, the sensor exhibited an ultrasensitive response toward the *lipL32* gene. As this is a sandwich type of assay, the response proportionally increased with the concentration of the *lipL32* gene, where a linear dynamic response (Figure 6) was observed in the 0−10 pg/mL range with a limit of detection (LOD) of 0.001 pg/mL (calculated from 3 SD_blank_/slope). Furthermore, the analytical performance of the CRIPSR-based dFnCas9 LFIA test strip exhibited the lowest LOD and widest linear dynamic range among the previously reported methods for the *lipL32* gene available so far. Therefore, the proposed CRIPSR-based dFnCas9 LFIA test strip confirms a strong potential for real sample analysis. In addition, the reproducibility of the test strip (the % relative standard deviation (RSD), n = 3) of the present method was examined. It was observed that the reproducibility for detecting the *lipL32* gene was lower than 5%, which is highly accepted for LFIA-based point-of-care techniques. This value showed that the newly developed immunosensor has a remarkably very low standard deviation with excellent fabrication repeatability. To demonstrate the stability of the immunosensor, the storage lifetime was then investigated. For the stability test of the strip, the new CRIPSR-based LFIA sensor was stored in a desiccator at room temperature for the stipulated time until use. The strips were stable for up to 2 weeks, with the percentage change in the signal less than 5% (% RSD = 4.31). This result indicated that the developed CRIPSR-based LFIA sensor demonstrated very satisfactory stability.

### 3.4. Specificity

**Figure 7:**
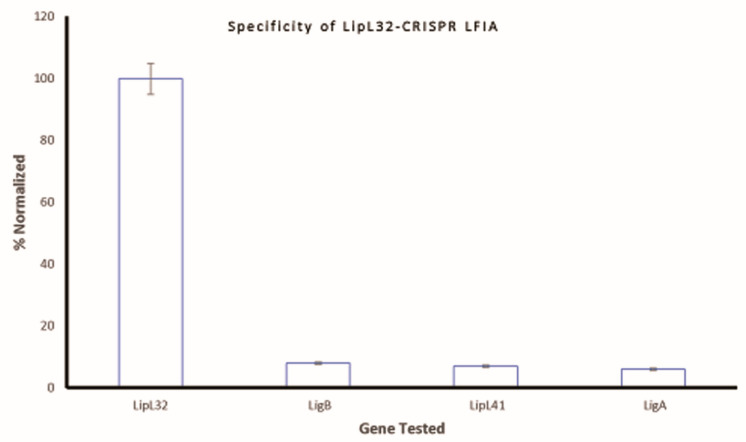
Specificity of the *Leptospira* lateral flow assay: *lipL32*, *ligB*, *lipL41*, *ligA*.

The cross-reactivity of the CRIPSR-based LFIA test strip was investigated for the non-specific reactions to other genes coding proteins/antigens. Three unrelated genes of *Leptospira* (*ligB*, *lipL41*, and *ligA*) were tested with the CRIPSR-based dFnCas9 LFIA test strip. As expected, the results were positive for the pathogenic *lipL32* gene only, while negligible responses were obtained for the other genes tested (Figure 7). Therefore, it can be concluded that the developed CRIPSR-based LFIA test strip sensor has excellent specificity to *lipL32.*

## 4. Conclusions

PCR is the gold standard for the specific and sensitive quantification of many pathogens. However, due to the requirement of sophisticated instrumentation and the relatively high cost of the technique when compared to that needed for rapid point-of-care tests, PCR has not been widely used as an early diagnostic tool for *Leptospira*. The CRISPR-based dFnCas9 LFIA test strip is a new nucleic acid detection platform able to diagnose many infectious diseases. This study is the first report for *Leptospira* detection using the dFnCas9 LFIA assay targeting the *lipL32* gene.

In this study, we developed a dFnCas9 lateral flow immunoassay for diagnosing leptospirosis targeting *Leptospira lipL32* gene detection. This lateral flow test strip was made portable and thus allowed for on-field testing. Interestingly, the developed test strip exhibited a LOD of 1 fg/mL with a linear dynamic range between 0 and 10 pg/mL, the lowest LOD among other reports available thus far. In summary, we believe that this developed lateral flow platform can be further extended for other biomarker detection, whereby a sensitive yet straightforward approach is of primary concern. The developed test strip can be used as a sensitive approach to diagnosing leptospirosis using DNA extracted from clinical samples from patients and samples from animals associated with the reproductive disorder.

## Figures and Tables

**Table 1:** Oligonucleotides were used for the development of the dFnCas9 lateral flow assay.

Primer	Dir	Sequence
*lipL32* gene	F	B-GAA GTG AAA GGA TCT TTC GTT GCA
*lipL32* gene	R	B-CGT CAG AAG CAG CTT TTT TCA AAG
*lipL32* gene	F	B-GGT ATT CCA GGT GTG AGC CC
*lipL32* gene	R	B-CGC GTC AGA AGC AGC TTT TT
crRNA IVT DNA oligo	F	5′ TAA TAC GAC TCA CTA TAC TCA AAT CCT GAA GAA TTG CGT TTC AGT TGC TGA ATT AT 3′
crRNA IVT DNA oligo	R	5′ ATA ATT CAG CAA CTG AAA CGC AAT TCT TCA GGA TTT GAG TAT AGT GAG TCG TAT TA 3′
dFnCas9-Syn-tracrRNA		5′ G*U*A AUU AAU GCU CUG UAA UCA UUU AAA AGU AUU UUG AAC GGA CCU CUG UUU GAC ACG UC*U* U – FITC 3′

## Data Availability

Data are available from corresponding authors, which can be obtained on formal request.

## Supplementary Materials

The following supporting information can be downloaded at: https://www.mdpi.com/article/10.3390/s23146544/s1.
