# Supplementary material for: A Lateral Flow Assay for the Detection of Leptospira lipL32 Gene Using CRISPR Technology
Source: Sensors (Basel). 2023 Jul 20;23(14):6544. doi: 10.3390/s23146544 (PMC10385553; doi:10.3390/s23146544)
Supplement: Supplementary file 1 [file sensors-23-06544-s001.zip › sensors-2493988-supplementary.pdf]

Supplementary Information

A Lateral Flow Assay for the Detection of *Leptospira lipL32* Gene Using CRISPR Technology

Satheesh Natarajan [1,*], Jayaraj Joseph [2], Balamurugan Vinayagamurthy [3] and Pedro Estrela [4,5,]

[1] Healthcare Technology Innovation Centre, Indian Institute of Technology Madras, Chennai 600113, India
[2] Department of Electrical Engineering, Indian Institute of Technology Madras, Chennai 600036, India; jayaraj@ee.iitm.ac.in
[3] Indian Council of Agricultural Research-National Institute of Veterinary Epidemiology and Disease Informatics (ICAR-NIVEDI), Bangalore 560064, India; b.vinayagamurthy@icar.gov.in
[4] Department of Electronic and Electrical Engineering, University of Bath, Bath BA2 7AY, UK; p.estrela@bath.ac.uk
[5] Centre for Bioengineering & Biomedical Technologies, University of Bath, Bath BA2 7AY, UK
* Correspondence: satheesh@htic.iitm.ac.in

Protocol for CRISPR/Cas-based LFIA for *lipL32* gene

1. **Preparation of CRISPR-Cas9 crRNAs**

Reagents	Volume	Final concentration
Forward Oligo (100 mM)	1.25 µL	2.5 µM
Reverse Oligo (100 mM)	1.25 µL	2.5 µM
Total volume	Up to 50 µL	

Synthesis of *in vitro* transcribed (IVT) crRNAs targeting the *lipL32* gene

To assemble the equimolar ratio of Forward and Reverse oligos (refer to the table above) for each target:

- Heat the reaction mix at 95°C for 5 minutes, followed by slow cooling at room temperature for 15 minutes.
- Perform *in vitro* transcription using a commercially available T7 Polymerase based IVT kit as per recommended protocol.

HI Scribe™ T7 High Yield RNA Synthesis Kit NEB #E2040S

Reagent	Amount (µL)	Concentration
Nuclease-free water	8.5	-
10× Reaction Buffer	1.5	0.75×
ATP (MM)	1.5	7.5 mM

GTP (MM)	1.5	7.5 mM
UTP (MM)	1.5	7.5 mM
CTP (MM)	1.5	7.5 mM
Template DNA	2	1 µg
T7 RNA Polymerase Mix	2	10 mM
Total Reaction Volume	20	

- Incubate at 37°C for 16 hrs (overnight).
- Add 1 ml of Turbo DNase to the reaction mixes and incubate at 37°C for 30 minutes.
- Heat inactivates at 70°C for 10 min.
- Optional: RNA can be visualised on a 2% agarose gel to check its integrity.

Column-based RNA clean-up as per the commercially provided protocols (Monarch® RNA Clean-up Kit Protocol (NEB #T2050S**)**

2. Generation of chimeric gRNAs (crRNA:tracrRNA-FITC).

**Reagents**	**Volume (µL)**	**Final concentration**
IVT synthesised crRNA	5	1 µM
FITC labelled tracrRNA	5	1 µM
Annealing Buffer<br><br>(100 mM NaCl, 50 mM Tris-Cl pH 8.0, 1 mM MgCl2)	Up to 50µL	

Heat the reaction mix at 95°C for 5 minutes, followed by slow cooling at room temperature for 15 minutes.

3. <u>CRISPR/Cas-based detection for *lipL32* gene by PCR</u>

- *Leptospira* DNA.
- Set up a single-step PCR reaction.

Reagents	Volume (µL)
Forward Biotinylated Primer (10 mM)	1
Reverse Biotinylated Primer (10 mM)	1
Master mix	12.5
DNA sample (5 ng)	1
Total Volume (with nuclease-free water)	20

Reaction conditions

Initial denaturation	95	1min
Denaturation	95	5 Sec
Annealing	60	10Sec
Polymerization	72	10Sec
Final Poly	72	1min
Cycle	40	
Infinite		

4. **Prepare dFnCas9-chimeric gRNA-RNP complexes for the samples to be tested.**

RNP complex against the *lipL-32* gene should be assembled for each sample. Incubate dFnCas9 protein with Chimeric FITC-labelled guides RNA to generate RNP complexes for 10 min at Room Temperature (RT).

Reagents	Volume (µL)	Final concentration
dFnCas9 protein (1 µM)	1	100 nM
Chimeric FITC-labelled gRNA (1 µM)	1	100 nM
Total Volume (Buffer containing 20 mM HEPES pH 7.5, 150mM KCl, 10% glycerol, 1mM DTT and 10 mM $MgCl_2$)	Up to 5	

- Add 5 µL of the biotinylated amplicon (from Step 3) to 5 µL of the dFnCas9-chimeric gRNA-RNP complex (Step 5).
- Incubate the reaction mix (containing RNP complex and amplicon) at 37°C for 10 min in a heating block or water bath.
- Add 90 µL of Lateral flow chase buffer to each tube containing 10 µL of the reaction mix from the previous step.
- After 15 minutes Insert the lateral flow strip directly into reaction tubes.
- Detect with the iQuant Instrument

Figure SI

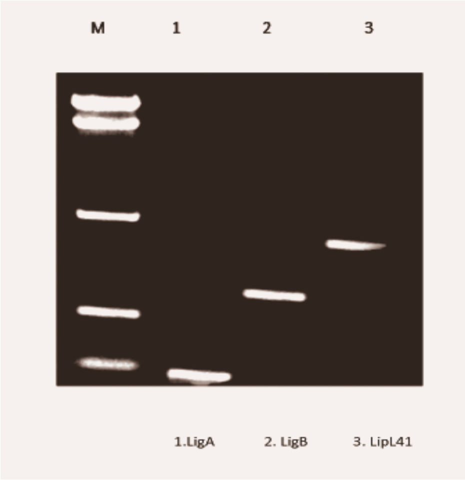

PCR for the *lig A, lig B, lipL41* and *lipL32* and the primers

			Size Bp
lig A	F	GCATA C CAT GG CGTC CTC TAA TAC GGA TAT	1491
	R	ATA CTCGAG CGT AAC TGG AGT ATA AGA ACT CT	
lig B	F	ATATCCGGAATGAATTTTGGTGTAAA	1041
	R	ATTTTCAAGATTTGTTCTCCAGATTT	
lipL41	F	GACCTCAGTAAACGCGCCGATAT	427
	R	CAGCGGCTTCGTCCAATCCT	
